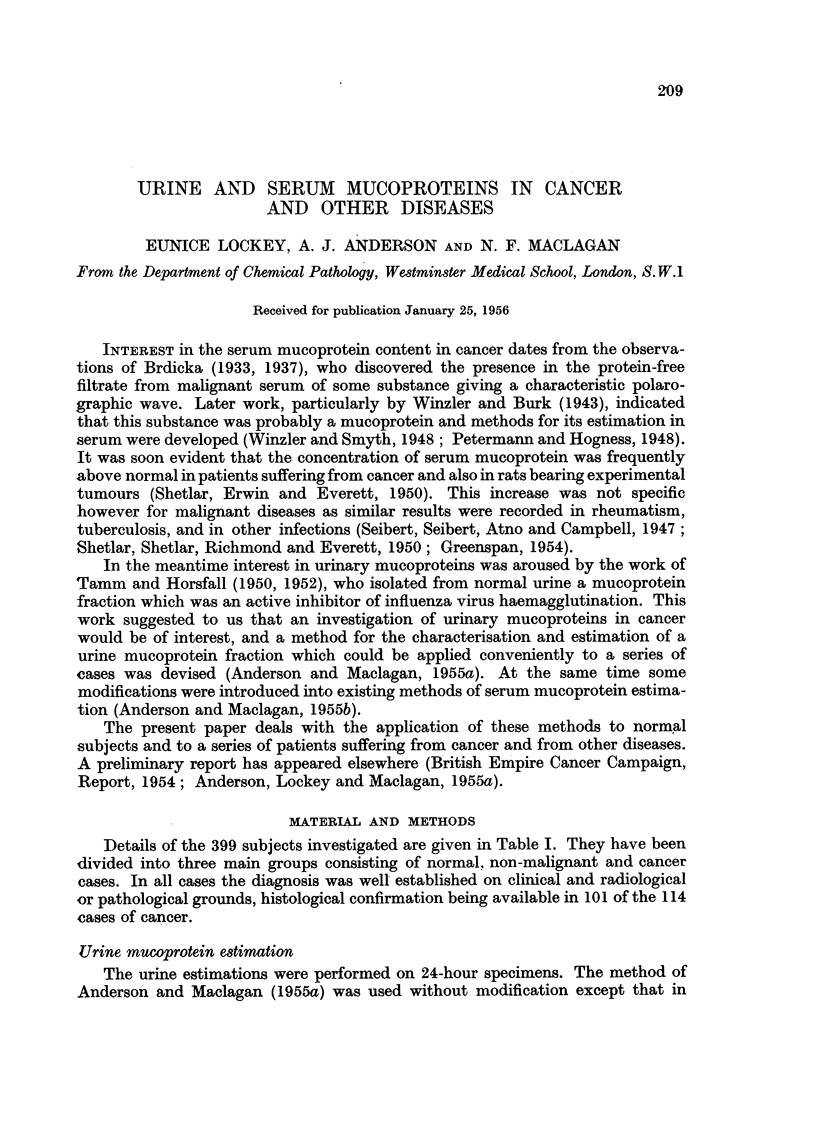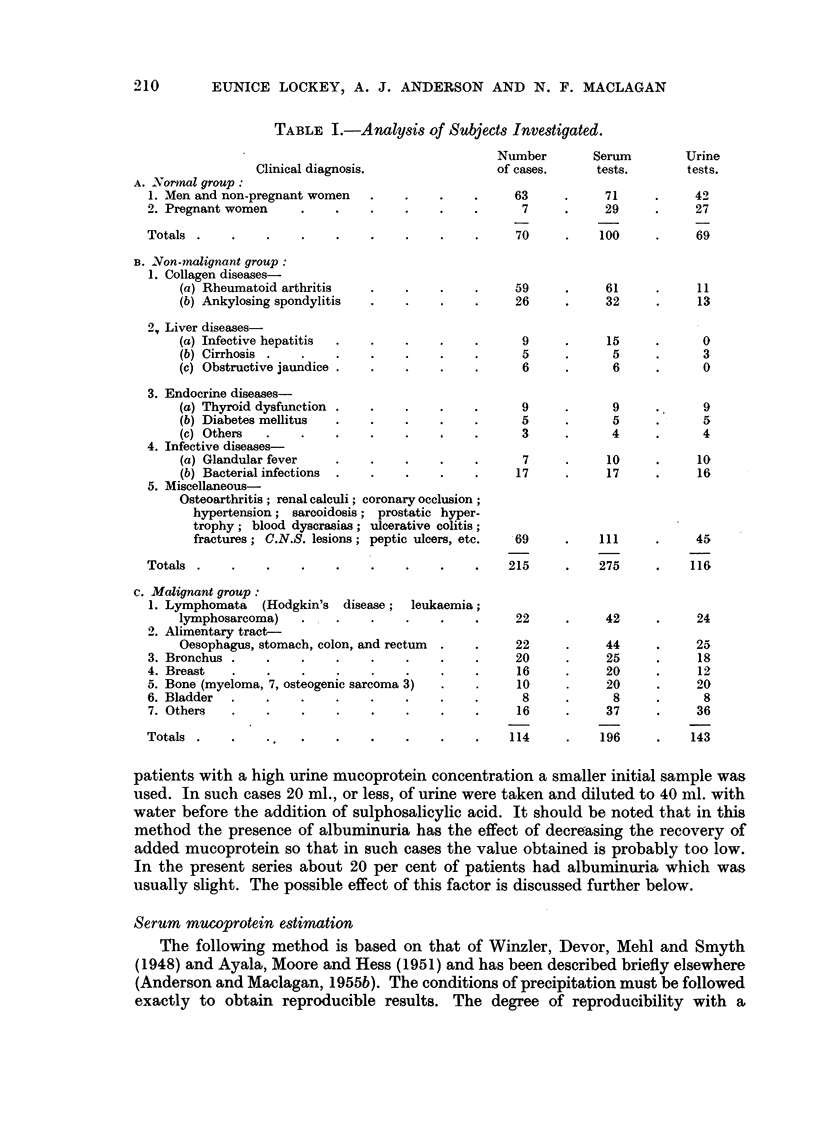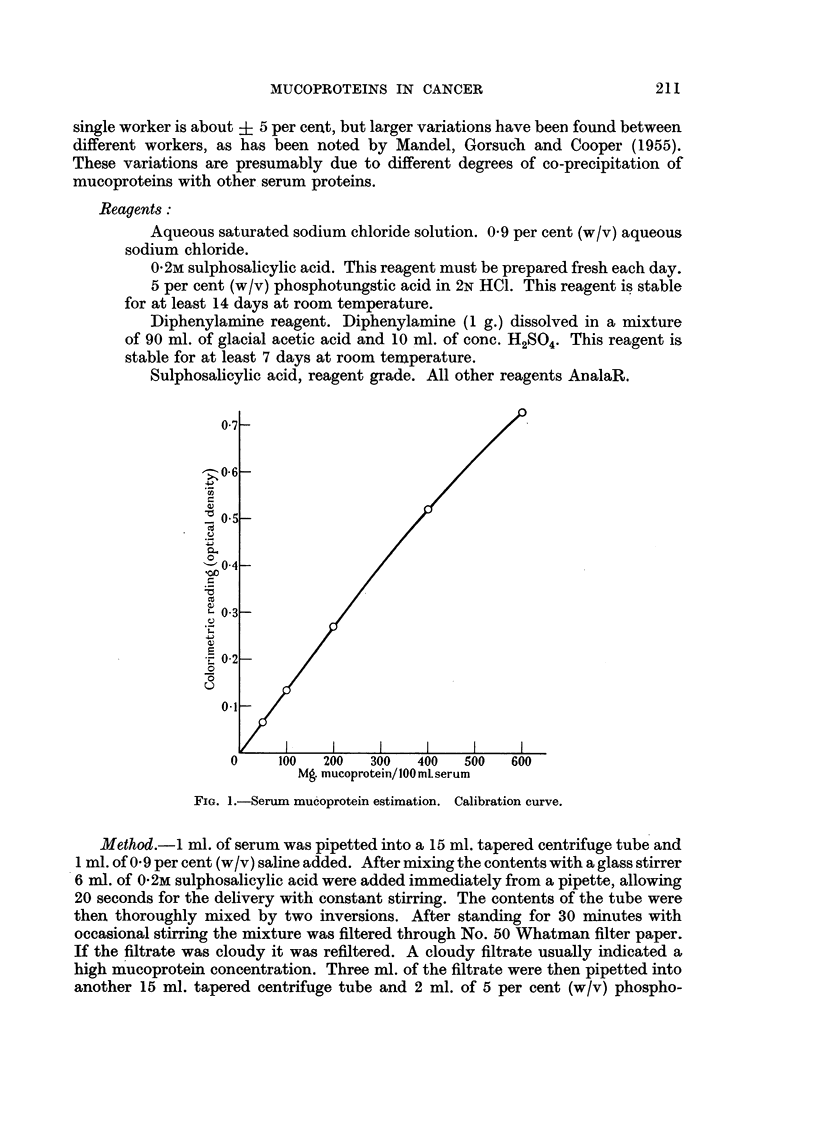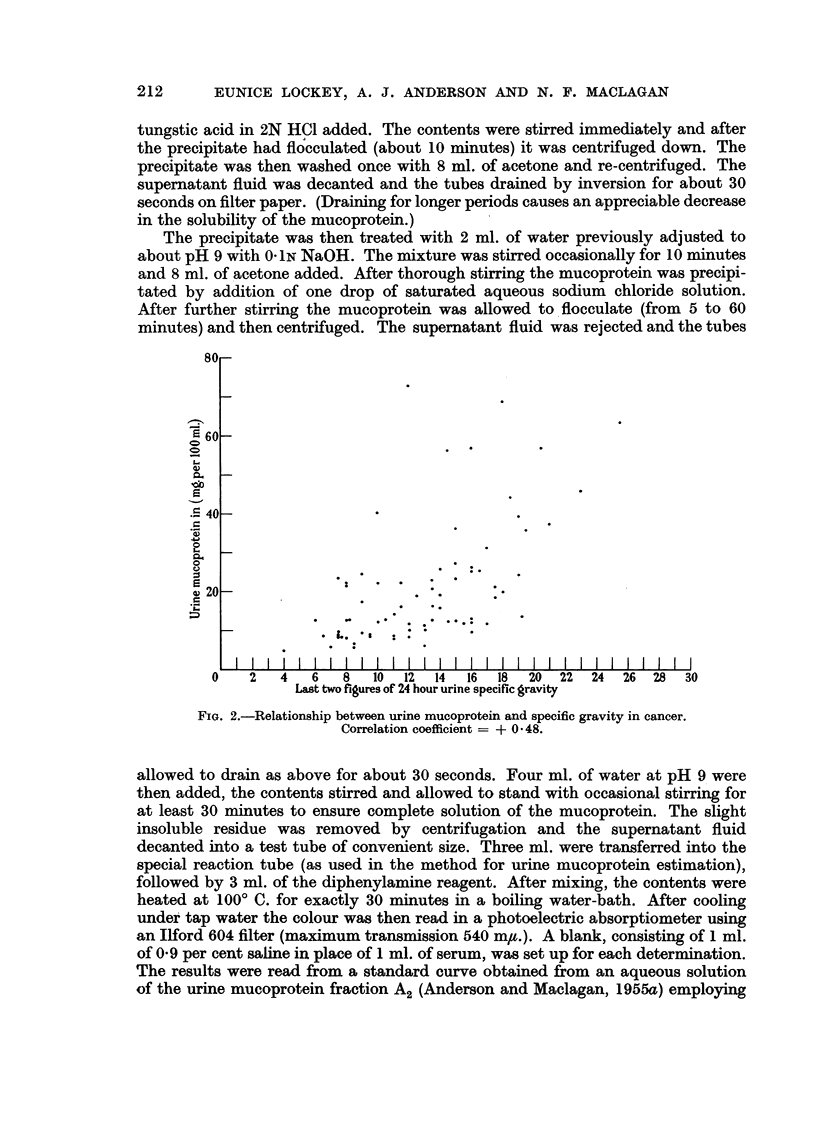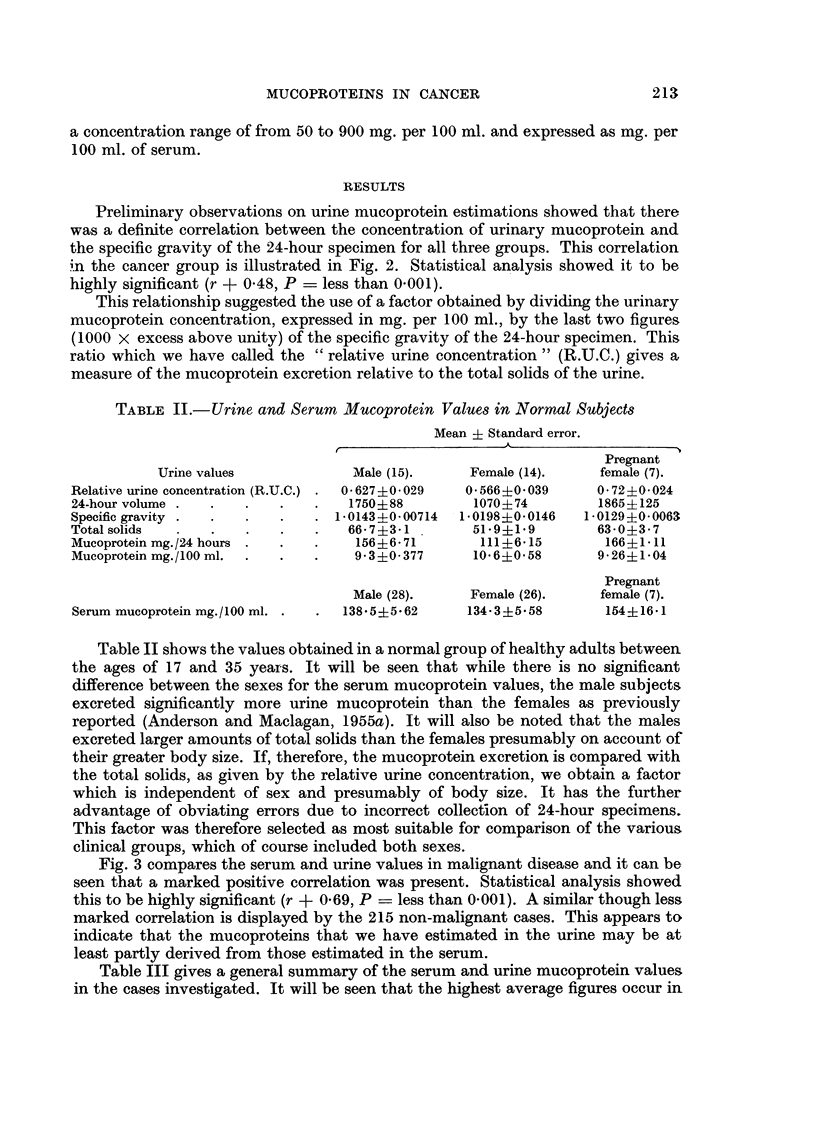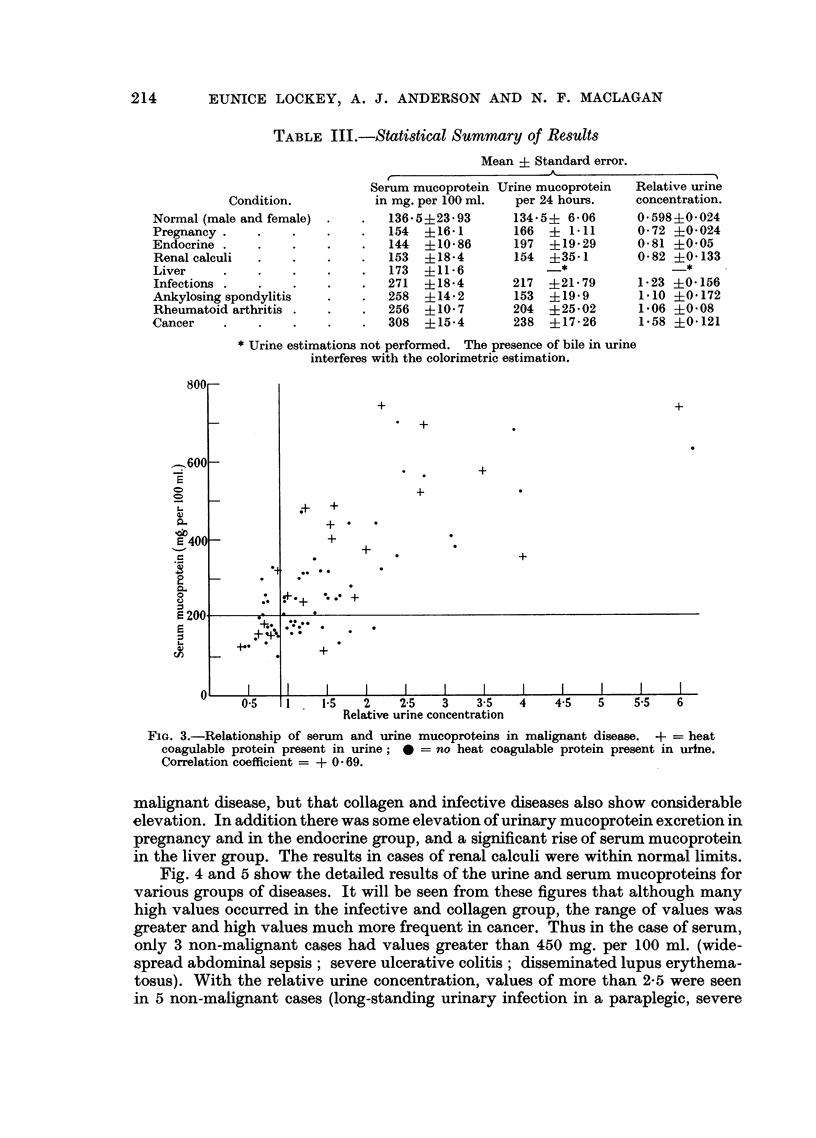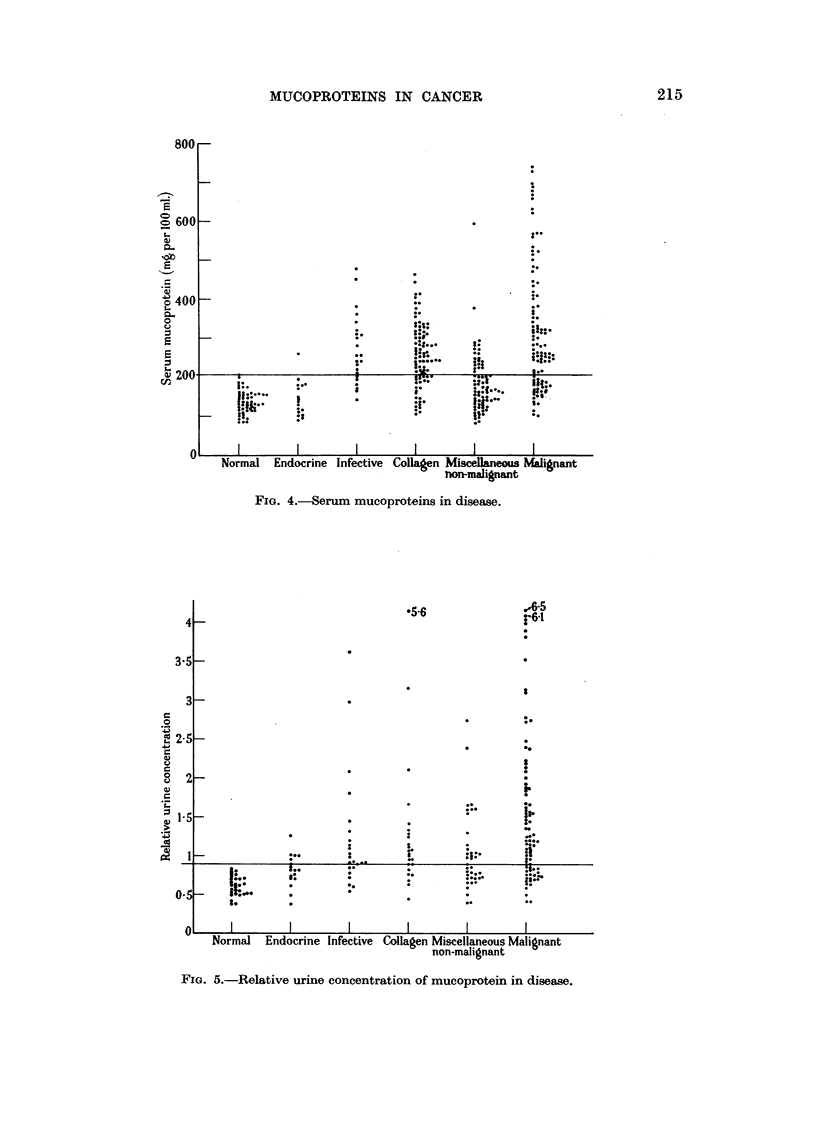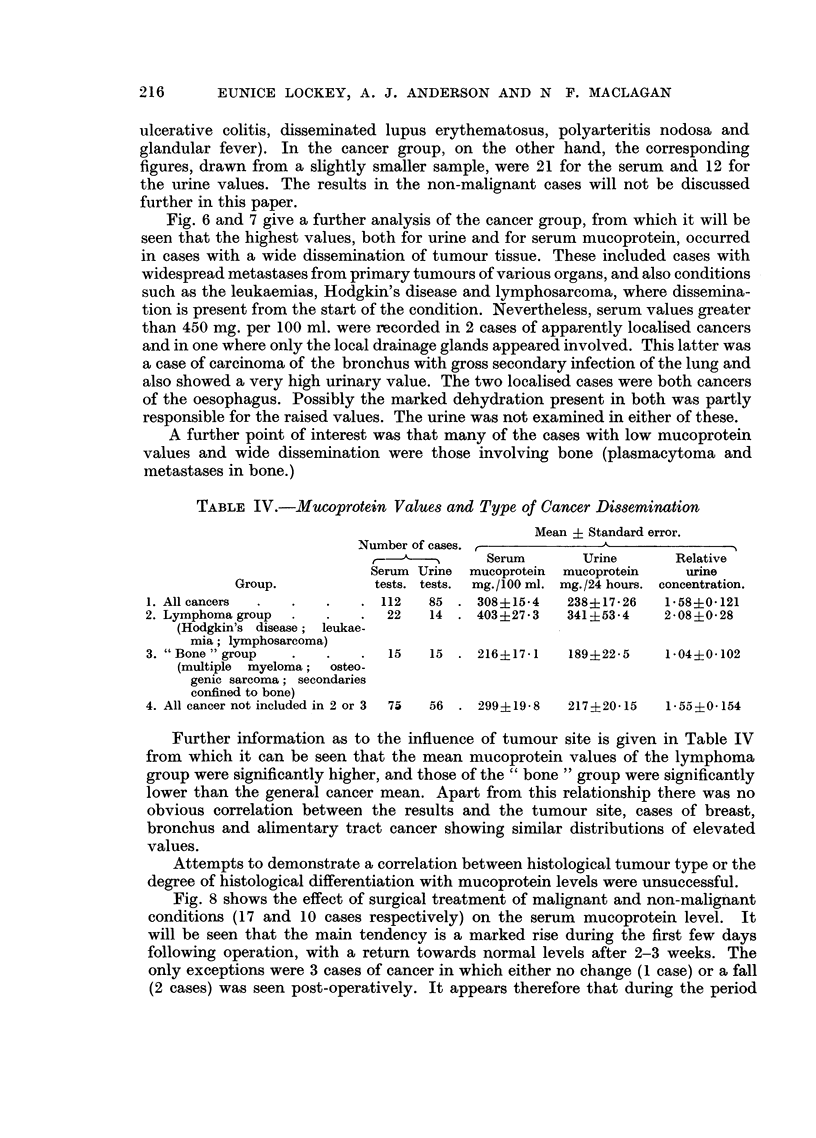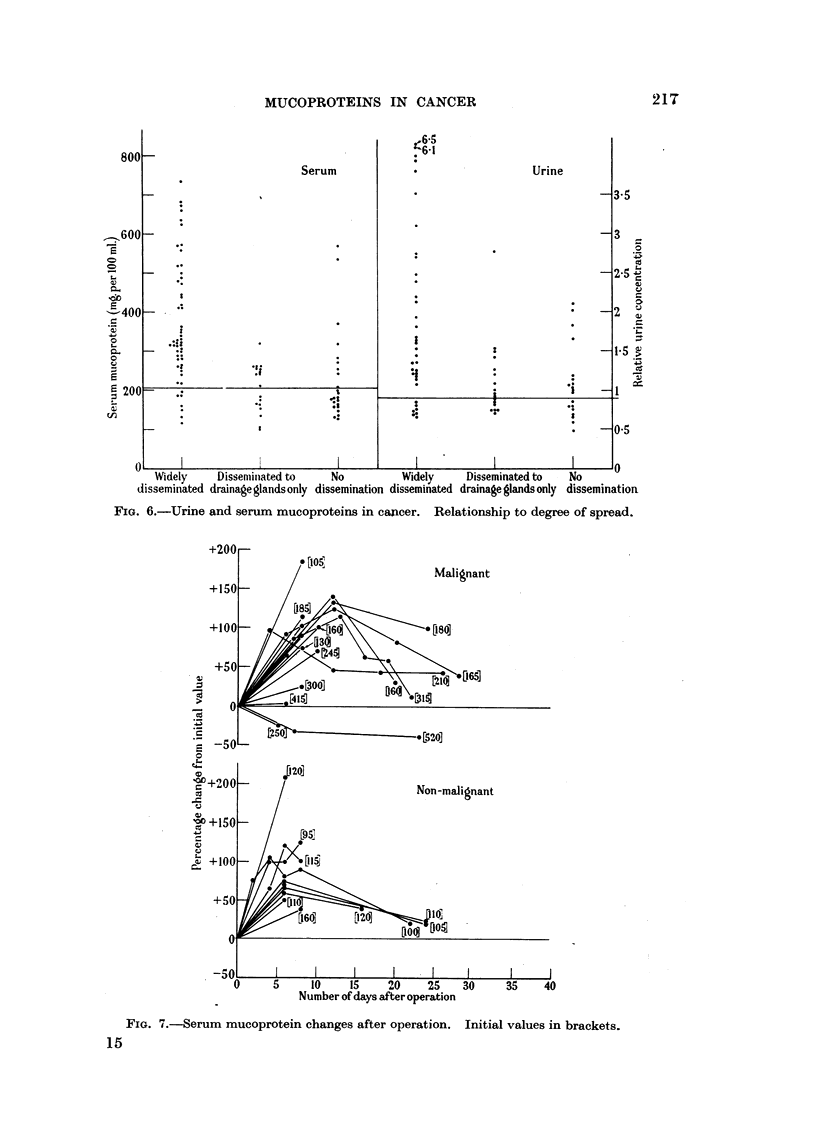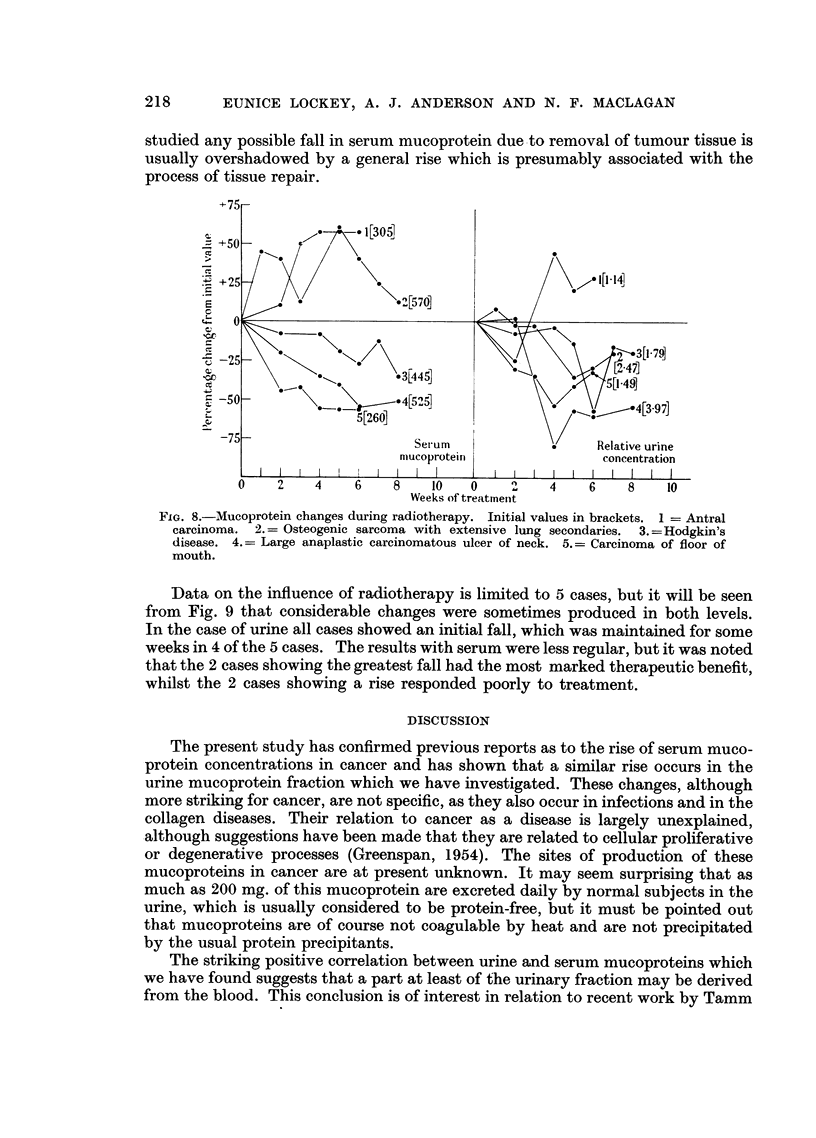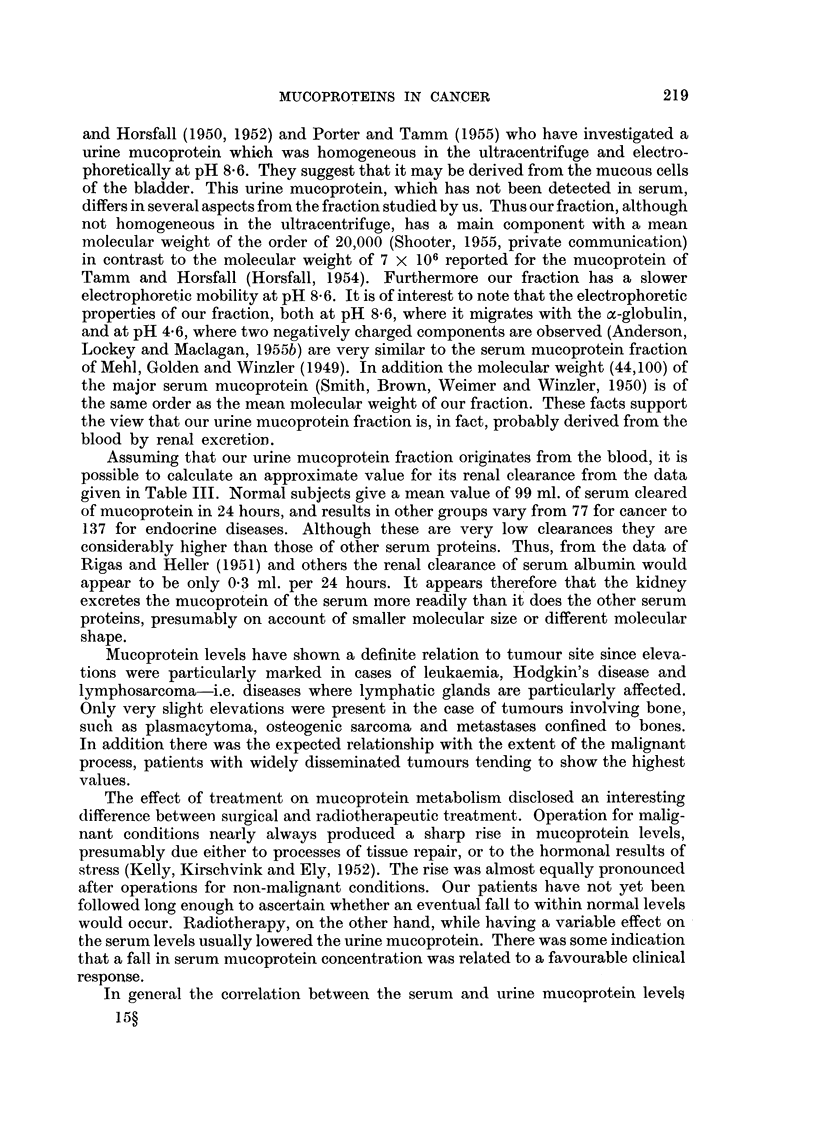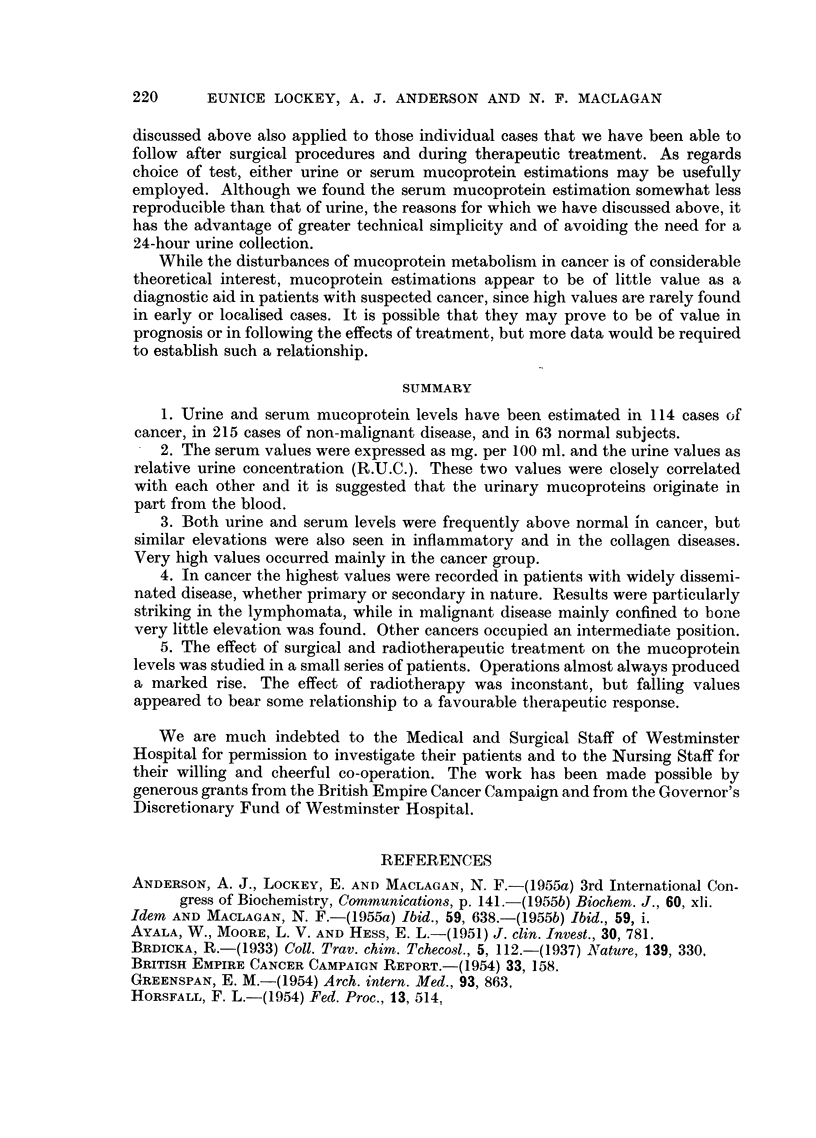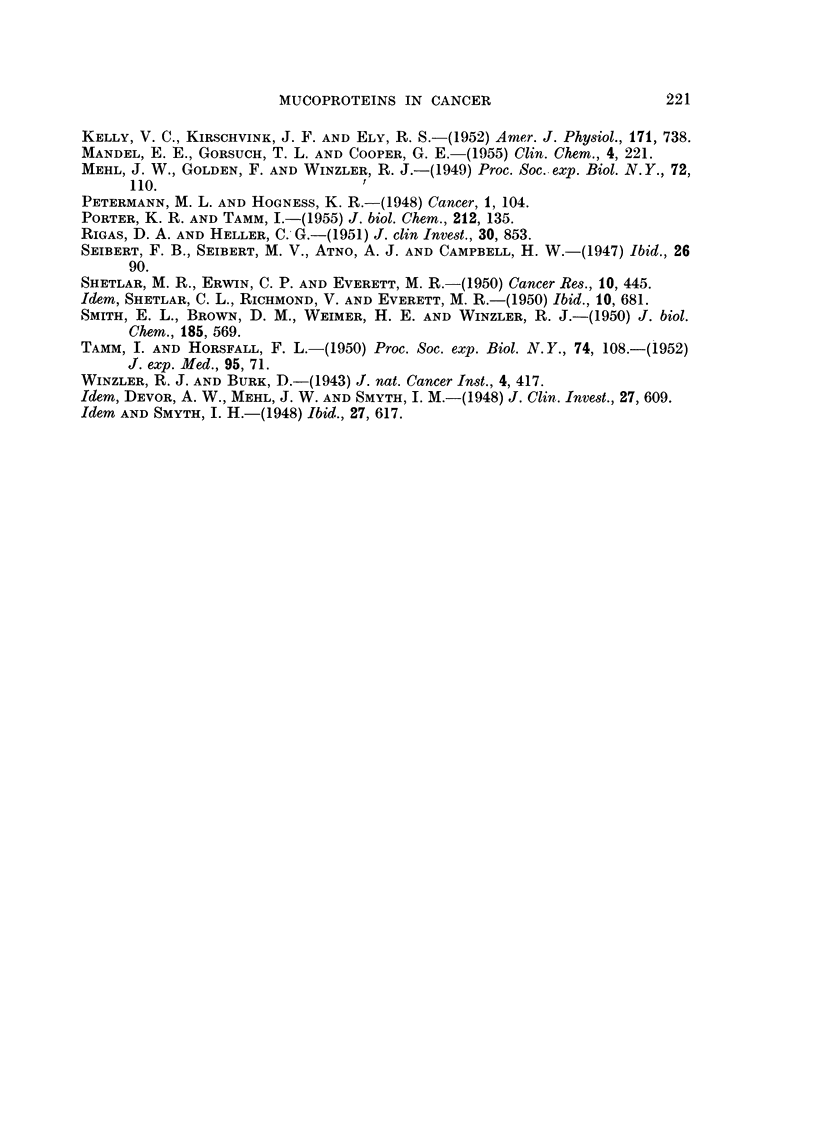# Urine and Serum Mucoproteins in Cancer and other Diseases

**DOI:** 10.1038/bjc.1956.24

**Published:** 1956-03

**Authors:** Eunice Lockey, A. J. Anderson, N. F. Maclagan


### URINE AND SERUM MUCOPROTEINS IN CANCER AND OTHER DISEASES

EUNICE LOCKEY, A. J. ANDERSON AND N. F. MACLAGAN

*From the Department of Chemical Pathology, Westminster Medical School, London, S.W.1*

Received for publication January 25, 1956

INTEREST in the serum mucoprotein content in cancer dates from the observations of Brdicka (1933, 1937), who discovered the presence in the protein-free filtrate from malignant serum of some substance giving a characteristic polarographic wave. Later work, particularly by Winzler and Burk (1943), indicated that this substance was probably a mucoprotein and methods for its estimation in serum were developed (Winzler and Smyth, 1948 ; Petermann and Hogness, 1948). It was soon evident that the concentration of serum mucoprotein was frequently above normal in patients suffering from cancer and also in rats bearing experimental tumours (Shetlar, Erwin and Everett, 1950). This increase was not specific however for malignant diseases as similar results were recorded in rheumatism, tuberculosis, and in other infections (Seibert, Seibert, Atno and Campbell, 1947 ; Shetlar, Shetlar, Richmond and Everett, 1950 ; Greenspan, 1954).

In the meantime interest in urinary mucoproteins was aroused by the work of Tamm and Horsfall (1950, 1952), who isolated from normal urine a mucoprotein fraction which was an active inhibitor of influenza virus haemagglutination. This work suggested to us that an investigation of urinary mucoproteins in cancer would be of interest, and a method for the characterisation and estimation of a urine mucoprotein fraction which could be applied conveniently to a series of cases was devised (Anderson and Maclagan, 1955*a*). At the same time some modifications were introduced into existing methods of serum mucoprotein estimation (Anderson and Maclagan, 1955*b*).

The present paper deals with the application of these methods to normal subjects and to a series of patients suffering from cancer and from other diseases. A preliminary report has appeared elsewhere (British Empire Cancer Campaign, Report, 1954 ; Anderson, Lockey and Maclagan, 1955*a*).

### MATERIAL AND METHODS

Details of the 399 subjects investigated are given in Table I. They have been divided into three main groups consisting of normal, non-malignant and cancer cases. In all cases the diagnosis was well established on clinical and radiological or pathological grounds, histological confirmation being available in 101 of the 114 cases of cancer.

### *Urine mucoprotein estimation*

The urine estimations were performed on 24-hour specimens. The method of Anderson and Maclagan (1955*a*) was used without modification except that in

TABLE I.—*Analysis of Subjects Investigated.*

| Clinical diagnosis. | Number of cases. | Serum tests. | Urine tests. |
|---|---|---|---|
| A. *Normal group :* | | | |
| 1. Men and non-pregnant women | 63 | 71 | 42 |
| 2. Pregnant women | 7 | 29 | 27 |
| Totals | 70 | 100 | 69 |
| B. *Non-malignant group :* | | | |
| 1. Collagen diseases— | | | |
| (*a*) Rheumatoid arthritis | 59 | 61 | 11 |
| (*b*) Ankylosing spondylitis | 26 | 32 | 13 |
| 2. Liver diseases— | | | |
| (*a*) Infective hepatitis | 9 | 15 | 0 |
| (*b*) Cirrhosis | 5 | 5 | 3 |
| (*c*) Obstructive jaundice | 6 | 6 | 0 |
| 3. Endocrine diseases— | | | |
| (*a*) Thyroid dysfunction | 9 | 9 | 9 |
| (*b*) Diabetes mellitus | 5 | 5 | 5 |
| (*c*) Others | 3 | 4 | 4 |
| 4. Infective diseases— | | | |
| (*a*) Glandular fever | 7 | 10 | 10 |
| (*b*) Bacterial infections | 17 | 17 | 16 |
| 5. Miscellaneous— | | | |
| Osteoarthritis ; renal calculi ; coronary occlusion ; hypertension ; sarcoidosis ; prostatic hypertrophy ; blood dyscrasias ; ulcerative colitis ; fractures ; *C.N.S.* lesions ; peptic ulcers, etc. | 69 | 111 | 45 |
| Totals | 215 | 275 | 116 |
| C. *Malignant group :* | | | |
| 1. Lymphomata (Hodgkin's disease ; leukaemia ; lymphosarcoma) | 22 | 42 | 24 |
| 2. Alimentary tract— Oesophagus, stomach, colon, and rectum | 22 | 44 | 25 |
| 3. Bronchus | 20 | 25 | 18 |
| 4. Breast | 16 | 20 | 12 |
| 5. Bone (myeloma, 7, osteogenic sarcoma 3) | 10 | 20 | 20 |
| 6. Bladder | 8 | 8 | 8 |
| 7. Others | 16 | 37 | 36 |
| Totals | 114 | 196 | 143 |

patients with a high urine mucoprotein concentration a smaller initial sample was used. In such cases 20 ml., or less, of urine were taken and diluted to 40 ml. with water before the addition of sulphosalicylic acid. It should be noted that in this method the presence of albuminuria has the effect of decreasing the recovery of added mucoprotein so that in such cases the value obtained is probably too low. In the present series about 20 per cent of patients had albuminuria which was usually slight. The possible effect of this factor is discussed further below.

*Serum mucoprotein estimation*

The following method is based on that of Winzler, Devor, Mehl and Smyth (1948) and Ayala, Moore and Hess (1951) and has been described briefly elsewhere (Anderson and Maclagan, 1955*b*). The conditions of precipitation must be followed exactly to obtain reproducible results. The degree of reproducibility with a single worker is about $\pm$ 5 per cent, but larger variations have been found between different workers, as has been noted by Mandel, Gorsuch and Cooper (1955). These variations are presumably due to different degrees of co-precipitation of mucoproteins with other serum proteins.

*Reagents :*

Aqueous saturated sodium chloride solution. 0·9 per cent (w/v) aqueous sodium chloride.

0·2M sulphosalicylic acid. This reagent must be prepared fresh each day.

5 per cent (w/v) phosphotungstic acid in 2N HCl. This reagent is stable for at least 14 days at room temperature.

Diphenylamine reagent. Diphenylamine (1 g.) dissolved in a mixture of 90 ml. of glacial acetic acid and 10 ml. of conc. $H_2SO_4$. This reagent is stable for at least 7 days at room temperature.

Sulphosalicylic acid, reagent grade. All other reagents AnalaR.

FIG. 1.—Serum mucoprotein estimation. Calibration curve.

*Method.*—1 ml. of serum was pipetted into a 15 ml. tapered centrifuge tube and 1 ml. of 0·9 per cent (w/v) saline added. After mixing the contents with a glass stirrer 6 ml. of 0·2M sulphosalicylic acid were added immediately from a pipette, allowing 20 seconds for the delivery with constant stirring. The contents of the tube were then thoroughly mixed by two inversions. After standing for 30 minutes with occasional stirring the mixture was filtered through No. 50 Whatman filter paper. If the filtrate was cloudy it was refiltered. A cloudy filtrate usually indicated a high mucoprotein concentration. Three ml. of the filtrate were then pipetted into another 15 ml. tapered centrifuge tube and 2 ml. of 5 per cent (w/v) phosphotungstic acid in 2N HCl added. The contents were stirred immediately and after the precipitate had flocculated (about 10 minutes) it was centrifuged down. The precipitate was then washed once with 8 ml. of acetone and re-centrifuged. The supernatant fluid was decanted and the tubes drained by inversion for about 30 seconds on filter paper. (Draining for longer periods causes an appreciable decrease in the solubility of the mucoprotein.)

The precipitate was then treated with 2 ml. of water previously adjusted to about pH 9 with 0·1N NaOH. The mixture was stirred occasionally for 10 minutes and 8 ml. of acetone added. After thorough stirring the mucoprotein was precipitated by addition of one drop of saturated aqueous sodium chloride solution. After further stirring the mucoprotein was allowed to flocculate (from 5 to 60 minutes) and then centrifuged. The supernatant fluid was rejected and the tubes

FIG. 2.—Relationship between urine mucoprotein and specific gravity in cancer. Correlation coefficient = + 0·48.

allowed to drain as above for about 30 seconds. Four ml. of water at pH 9 were then added, the contents stirred and allowed to stand with occasional stirring for at least 30 minutes to ensure complete solution of the mucoprotein. The slight insoluble residue was removed by centrifugation and the supernatant fluid decanted into a test tube of convenient size. Three ml. were transferred into the special reaction tube (as used in the method for urine mucoprotein estimation), followed by 3 ml. of the diphenylamine reagent. After mixing, the contents were heated at 100° C. for exactly 30 minutes in a boiling water-bath. After cooling under tap water the colour was then read in a photoelectric absorptiometer using an Ilford 604 filter (maximum transmission 540 mμ.). A blank, consisting of 1 ml. of 0·9 per cent saline in place of 1 ml. of serum, was set up for each determination. The results were read from a standard curve obtained from an aqueous solution of the urine mucoprotein fraction $A_2$ (Anderson and Maclagan, 1955a) employing a concentration range of from 50 to 900 mg. per 100 ml. and expressed as mg. per 100 ml. of serum.

### RESULTS

Preliminary observations on urine mucoprotein estimations showed that there was a definite correlation between the concentration of urinary mucoprotein and the specific gravity of the 24-hour specimen for all three groups. This correlation in the cancer group is illustrated in Fig. 2. Statistical analysis showed it to be highly significant ($r + 0{\cdot}48$, $P =$ less than 0·001).

This relationship suggested the use of a factor obtained by dividing the urinary mucoprotein concentration, expressed in mg. per 100 ml., by the last two figures ($1000 \times$ excess above unity) of the specific gravity of the 24-hour specimen. This ratio which we have called the " relative urine concentration " (R.U.C.) gives a measure of the mucoprotein excretion relative to the total solids of the urine.

TABLE II.—*Urine and Serum Mucoprotein Values in Normal Subjects*

| | Mean ± Standard error. | | |
|---|---|---|---|
| Urine values | Male (15). | Female (14). | Pregnant female (7). |
| Relative urine concentration (R.U.C.) | 0·627±0·029 | 0·566±0·039 | 0·72±0·024 |
| 24-hour volume | 1750±88 | 1070±74 | 1865±125 |
| Specific gravity | 1·0143±0·00714 | 1·0198±0·0146 | 1·0129±0·0063 |
| Total solids | 66·7±3·1 | 51·9±1·9 | 63·0±3·7 |
| Mucoprotein mg./24 hours | 156±6·71 | 111±6·15 | 166±1·11 |
| Mucoprotein mg./100 ml. | 9·3±0·377 | 10·6±0·58 | 9·26±1·04 |
| | Male (28). | Female (26). | Pregnant female (7). |
| Serum mucoprotein mg./100 ml. | 138·5±5·62 | 134·3±5·58 | 154±16·1 |

Table II shows the values obtained in a normal group of healthy adults between the ages of 17 and 35 years. It will be seen that while there is no significant difference between the sexes for the serum mucoprotein values, the male subjects excreted significantly more urine mucoprotein than the females as previously reported (Anderson and Maclagan, 1955*a*). It will also be noted that the males excreted larger amounts of total solids than the females presumably on account of their greater body size. If, therefore, the mucoprotein excretion is compared with the total solids, as given by the relative urine concentration, we obtain a factor which is independent of sex and presumably of body size. It has the further advantage of obviating errors due to incorrect collection of 24-hour specimens. This factor was therefore selected as most suitable for comparison of the various clinical groups, which of course included both sexes.

Fig. 3 compares the serum and urine values in malignant disease and it can be seen that a marked positive correlation was present. Statistical analysis showed this to be highly significant ($r + 0{\cdot}69$, $P =$ less than 0·001). A similar though less marked correlation is displayed by the 215 non-malignant cases. This appears to indicate that the mucoproteins that we have estimated in the urine may be at least partly derived from those estimated in the serum.

Table III gives a general summary of the serum and urine mucoprotein values in the cases investigated. It will be seen that the highest average figures occur in

TABLE III.—*Statistical Summary of Results*

| Condition. | Serum mucoprotein in mg. per 100 ml. (Mean ± Standard error) | Urine mucoprotein per 24 hours. (Mean ± Standard error) | Relative urine concentration. (Mean ± Standard error) |
|---|---|---|---|
| Normal (male and female) | 136·5±23·93 | 134·5± 6·06 | 0·598±0·024 |
| Pregnancy | 154 ±16·1 | 166 ± 1·11 | 0·72 ±0·024 |
| Endocrine | 144 ±10·86 | 197 ±19·29 | 0·81 ±0·05 |
| Renal calculi | 153 ±18·4 | 154 ±35·1 | 0·82 ±0·133 |
| Liver | 173 ±11·6 | —* | —* |
| Infections | 271 ±18·4 | 217 ±21·79 | 1·23 ±0·156 |
| Ankylosing spondylitis | 258 ±14·2 | 153 ±19·9 | 1·10 ±0·172 |
| Rheumatoid arthritis | 256 ±10·7 | 204 ±25·02 | 1·06 ±0·08 |
| Cancer | 308 ±15·4 | 238 ±17·26 | 1·58 ±0·121 |

* Urine estimations not performed. The presence of bile in urine interferes with the colorimetric estimation.

FIG. 3.—Relationship of serum and urine mucoproteins in malignant disease. + = heat coagulable protein present in urine ; ● = *no* heat coagulable protein present in urine. Correlation coefficient = + 0·69.

malignant disease, but that collagen and infective diseases also show considerable elevation. In addition there was some elevation of urinary mucoprotein excretion in pregnancy and in the endocrine group, and a significant rise of serum mucoprotein in the liver group. The results in cases of renal calculi were within normal limits.

Fig. 4 and 5 show the detailed results of the urine and serum mucoproteins for various groups of diseases. It will be seen from these figures that although many high values occurred in the infective and collagen group, the range of values was greater and high values much more frequent in cancer. Thus in the case of serum, only 3 non-malignant cases had values greater than 450 mg. per 100 ml. (widespread abdominal sepsis ; severe ulcerative colitis ; disseminated lupus erythematosus). With the relative urine concentration, values of more than 2·5 were seen in 5 non-malignant cases (long-standing urinary infection in a paraplegic, severe

FIG. 4.—Serum mucoproteins in disease.

FIG. 5.—Relative urine concentration of mucoprotein in disease.

ulcerative colitis, disseminated lupus erythematosus, polyarteritis nodosa and glandular fever). In the cancer group, on the other hand, the corresponding figures, drawn from a slightly smaller sample, were 21 for the serum and 12 for the urine values. The results in the non-malignant cases will not be discussed further in this paper.

Fig. 6 and 7 give a further analysis of the cancer group, from which it will be seen that the highest values, both for urine and for serum mucoprotein, occurred in cases with a wide dissemination of tumour tissue. These included cases with widespread metastases from primary tumours of various organs, and also conditions such as the leukaemias, Hodgkin's disease and lymphosarcoma, where dissemination is present from the start of the condition. Nevertheless, serum values greater than 450 mg. per 100 ml. were recorded in 2 cases of apparently localised cancers and in one where only the local drainage glands appeared involved. This latter was a case of carcinoma of the bronchus with gross secondary infection of the lung and also showed a very high urinary value. The two localised cases were both cancers of the oesophagus. Possibly the marked dehydration present in both was partly responsible for the raised values. The urine was not examined in either of these.

A further point of interest was that many of the cases with low mucoprotein values and wide dissemination were those involving bone (plasmacytoma and metastases in bone.)

TABLE IV.—*Mucoprotein Values and Type of Cancer Dissemination*

| Group. | Number of cases. Serum tests. | Urine tests. | Mean ± Standard error. Serum mucoprotein mg./100 ml. | Urine mucoprotein mg./24 hours. | Relative urine concentration. |
|---|---|---|---|---|---|
| 1. All cancers | 112 | 85 | $308 \pm 15 \cdot 4$ | $238 \pm 17 \cdot 26$ | $1 \cdot 58 \pm 0 \cdot 121$ |
| 2. Lymphoma group (Hodgkin's disease; leukaemia; lymphosarcoma) | 22 | 14 | $403 \pm 27 \cdot 3$ | $341 \pm 53 \cdot 4$ | $2 \cdot 08 \pm 0 \cdot 28$ |
| 3. "Bone" group (multiple myeloma; osteogenic sarcoma; secondaries confined to bone) | 15 | 15 | $216 \pm 17 \cdot 1$ | $189 \pm 22 \cdot 5$ | $1 \cdot 04 \pm 0 \cdot 102$ |
| 4. All cancer not included in 2 or 3 | 75 | 56 | $299 \pm 19 \cdot 8$ | $217 \pm 20 \cdot 15$ | $1 \cdot 55 \pm 0 \cdot 154$ |

Further information as to the influence of tumour site is given in Table IV from which it can be seen that the mean mucoprotein values of the lymphoma group were significantly higher, and those of the "bone" group were significantly lower than the general cancer mean. Apart from this relationship there was no obvious correlation between the results and the tumour site, cases of breast, bronchus and alimentary tract cancer showing similar distributions of elevated values.

Attempts to demonstrate a correlation between histological tumour type or the degree of histological differentiation with mucoprotein levels were unsuccessful.

Fig. 8 shows the effect of surgical treatment of malignant and non-malignant conditions (17 and 10 cases respectively) on the serum mucoprotein level. It will be seen that the main tendency is a marked rise during the first few days following operation, with a return towards normal levels after 2–3 weeks. The only exceptions were 3 cases of cancer in which either no change (1 case) or a fall (2 cases) was seen post-operatively. It appears therefore that during the period

FIG. 6.—Urine and serum mucoproteins in cancer. Relationship to degree of spread.

FIG. 7.—Serum mucoprotein changes after operation. Initial values in brackets.

studied any possible fall in serum mucoprotein due to removal of tumour tissue is usually overshadowed by a general rise which is presumably associated with the process of tissue repair.

FIG. 8.—Mucoprotein changes during radiotherapy. Initial values in brackets. 1 = Antral carcinoma. 2. = Osteogenic sarcoma with extensive lung secondaries. 3. = Hodgkin's disease. 4. = Large anaplastic carcinomatous ulcer of neck. 5. = Carcinoma of floor of mouth.

Data on the influence of radiotherapy is limited to 5 cases, but it will be seen from Fig. 9 that considerable changes were sometimes produced in both levels. In the case of urine all cases showed an initial fall, which was maintained for some weeks in 4 of the 5 cases. The results with serum were less regular, but it was noted that the 2 cases showing the greatest fall had the most marked therapeutic benefit, whilst the 2 cases showing a rise responded poorly to treatment.

### DISCUSSION

The present study has confirmed previous reports as to the rise of serum mucoprotein concentrations in cancer and has shown that a similar rise occurs in the urine mucoprotein fraction which we have investigated. These changes, although more striking for cancer, are not specific, as they also occur in infections and in the collagen diseases. Their relation to cancer as a disease is largely unexplained, although suggestions have been made that they are related to cellular proliferative or degenerative processes (Greenspan, 1954). The sites of production of these mucoproteins in cancer are at present unknown. It may seem surprising that as much as 200 mg. of this mucoprotein are excreted daily by normal subjects in the urine, which is usually considered to be protein-free, but it must be pointed out that mucoproteins are of course not coagulable by heat and are not precipitated by the usual protein precipitants.

The striking positive correlation between urine and serum mucoproteins which we have found suggests that a part at least of the urinary fraction may be derived from the blood. This conclusion is of interest in relation to recent work by Tamm and Horsfall (1950, 1952) and Porter and Tamm (1955) who have investigated a urine mucoprotein which was homogeneous in the ultracentrifuge and electrophoretically at pH 8·6. They suggest that it may be derived from the mucous cells of the bladder. This urine mucoprotein, which has not been detected in serum, differs in several aspects from the fraction studied by us. Thus our fraction, although not homogeneous in the ultracentrifuge, has a main component with a mean molecular weight of the order of 20,000 (Shooter, 1955, private communication) in contrast to the molecular weight of $7 \times 10^6$ reported for the mucoprotein of Tamm and Horsfall (Horsfall, 1954). Furthermore our fraction has a slower electrophoretic mobility at pH 8·6. It is of interest to note that the electrophoretic properties of our fraction, both at pH 8·6, where it migrates with the $\alpha$-globulin, and at pH 4·6, where two negatively charged components are observed (Anderson, Lockey and Maclagan, 1955*b*) are very similar to the serum mucoprotein fraction of Mehl, Golden and Winzler (1949). In addition the molecular weight (44,100) of the major serum mucoprotein (Smith, Brown, Weimer and Winzler, 1950) is of the same order as the mean molecular weight of our fraction. These facts support the view that our urine mucoprotein fraction is, in fact, probably derived from the blood by renal excretion.

Assuming that our urine mucoprotein fraction originates from the blood, it is possible to calculate an approximate value for its renal clearance from the data given in Table III. Normal subjects give a mean value of 99 ml. of serum cleared of mucoprotein in 24 hours, and results in other groups vary from 77 for cancer to 137 for endocrine diseases. Although these are very low clearances they are considerably higher than those of other serum proteins. Thus, from the data of Rigas and Heller (1951) and others the renal clearance of serum albumin would appear to be only 0·3 ml. per 24 hours. It appears therefore that the kidney excretes the mucoprotein of the serum more readily than it does the other serum proteins, presumably on account of smaller molecular size or different molecular shape.

Mucoprotein levels have shown a definite relation to tumour site since elevations were particularly marked in cases of leukaemia, Hodgkin's disease and lymphosarcoma—i.e. diseases where lymphatic glands are particularly affected. Only very slight elevations were present in the case of tumours involving bone, such as plasmacytoma, osteogenic sarcoma and metastases confined to bones. In addition there was the expected relationship with the extent of the malignant process, patients with widely disseminated tumours tending to show the highest values.

The effect of treatment on mucoprotein metabolism disclosed an interesting difference between surgical and radiotherapeutic treatment. Operation for malignant conditions nearly always produced a sharp rise in mucoprotein levels, presumably due either to processes of tissue repair, or to the hormonal results of stress (Kelly, Kirschvink and Ely, 1952). The rise was almost equally pronounced after operations for non-malignant conditions. Our patients have not yet been followed long enough to ascertain whether an eventual fall to within normal levels would occur. Radiotherapy, on the other hand, while having a variable effect on the serum levels usually lowered the urine mucoprotein. There was some indication that a fall in serum mucoprotein concentration was related to a favourable clinical response.

In general the correlation between the serum and urine mucoprotein levels discussed above also applied to those individual cases that we have been able to follow after surgical procedures and during therapeutic treatment. As regards choice of test, either urine or serum mucoprotein estimations may be usefully employed. Although we found the serum mucoprotein estimation somewhat less reproducible than that of urine, the reasons for which we have discussed above, it has the advantage of greater technical simplicity and of avoiding the need for a 24-hour urine collection.

While the disturbances of mucoprotein metabolism in cancer is of considerable theoretical interest, mucoprotein estimations appear to be of little value as a diagnostic aid in patients with suspected cancer, since high values are rarely found in early or localised cases. It is possible that they may prove to be of value in prognosis or in following the effects of treatment, but more data would be required to establish such a relationship.

### SUMMARY

1. Urine and serum mucoprotein levels have been estimated in 114 cases of cancer, in 215 cases of non-malignant disease, and in 63 normal subjects.

2. The serum values were expressed as mg. per 100 ml. and the urine values as relative urine concentration (R.U.C.). These two values were closely correlated with each other and it is suggested that the urinary mucoproteins originate in part from the blood.

3. Both urine and serum levels were frequently above normal in cancer, but similar elevations were also seen in inflammatory and in the collagen diseases. Very high values occurred mainly in the cancer group.

4. In cancer the highest values were recorded in patients with widely disseminated disease, whether primary or secondary in nature. Results were particularly striking in the lymphomata, while in malignant disease mainly confined to bone very little elevation was found. Other cancers occupied an intermediate position.

5. The effect of surgical and radiotherapeutic treatment on the mucoprotein levels was studied in a small series of patients. Operations almost always produced a marked rise. The effect of radiotherapy was inconstant, but falling values appeared to bear some relationship to a favourable therapeutic response.

We are much indebted to the Medical and Surgical Staff of Westminster Hospital for permission to investigate their patients and to the Nursing Staff for their willing and cheerful co-operation. The work has been made possible by generous grants from the British Empire Cancer Campaign and from the Governor's Discretionary Fund of Westminster Hospital.

### REFERENCES

Anderson, A. J., Lockey, E. and Maclagan, N. F.—(1955*a*) 3rd International Congress of Biochemistry, *Communications*, p. 141.—(1955*b*) *Biochem. J.*, **60**, xli.

*Idem* and Maclagan, N. F.—(1955*a*) *Ibid.*, **59**, 638.—(1955*b*) *Ibid.*, **59**, i.

Ayala, W., Moore, L. V. and Hess, E. L.—(1951) *J. clin. Invest.*, **30**, 781.

Brdicka, R.—(1933) *Coll. Trav. chim. Tchecosl.*, **5**, 112.—(1937) *Nature*, **139**, 330.

British Empire Cancer Campaign Report.—(1954) **33**, 158.

Greenspan, E. M.—(1954) *Arch. intern. Med.*, **93**, 863.

Horsfall, F. L.—(1954) *Fed. Proc.*, **13**, 514.

KELLY, V. C., KIRSCHVINK, J. F. AND ELY, R. S.—(1952) *Amer. J. Physiol.*, **171**, 738.
MANDEL, E. E., GORSUCH, T. L. AND COOPER, G. E.—(1955) *Clin. Chem.*, **4**, 221.
MEHL, J. W., GOLDEN, F. AND WINZLER, R. J.—(1949) *Proc. Soc. exp. Biol. N.Y.*, **72**, 110.
PETERMANN, M. L. AND HOGNESS, K. R.—(1948) *Cancer*, **1**, 104.
PORTER, K. R. AND TAMM, I.—(1955) *J. biol. Chem.*, **212**, 135.
RIGAS, D. A. AND HELLER, C. G.—(1951) *J. clin Invest.*, **30**, 853.
SEIBERT, F. B., SEIBERT, M. V., ATNO, A. J. AND CAMPBELL, H. W.—(1947) *Ibid.*, **26** 90.
SHETLAR, M. R., ERWIN, C. P. AND EVERETT, M. R.—(1950) *Cancer Res.*, **10**, 445.
*Idem*, SHETLAR, C. L., RICHMOND, V. AND EVERETT, M. R.—(1950) *Ibid.*, **10**, 681.
SMITH, E. L., BROWN, D. M., WEIMER, H. E. AND WINZLER, R. J.—(1950) *J. biol. Chem.*, **185**, 569.
TAMM, I. AND HORSFALL, F. L.—(1950) *Proc. Soc. exp. Biol. N.Y.*, **74**, 108.—(1952) *J. exp. Med.*, **95**, 71.
WINZLER, R. J. AND BURK, D.—(1943) *J. nat. Cancer Inst.*, **4**, 417.
*Idem*, DEVOR, A. W., MEHL, J. W. AND SMYTH, I. M.—(1948) *J. Clin. Invest.*, **27**, 609.
*Idem* AND SMYTH, I. H.—(1948) *Ibid.*, **27**, 617.